# Predicting autologous hamstring graft diameter and finding reliable measurement levels in the Zhuang population using preoperative ultrasonography

**DOI:** 10.3389/fphys.2022.916438

**Published:** 2022-08-24

**Authors:** Xiao-Li Huang, Hong-Yu Zheng, Ze-Feng Shi, Hui-Hui Yang, Bing Zhang, Xiao-Chun Yang, Hong Wang, Ru-Xin Tan

**Affiliations:** ^1^ Department of Ultrasound, People’s Hospital of Guangxi Zhuang Autonomous Region, Guangxi Academy of Medical Sciences, Nanning, China; ^2^ Department of Orthopedic, People’s Hospital of Guangxi Zhuang Autonomous Region, Guangxi Academy of Medical Sciences, Nanning, China

**Keywords:** ACL reconstruction, autograft, Zhuang ethnic group, prediction, ultrasonography, semitendinosus tendon, gracilis tendon, diameter

## Abstract

**Purpose:** To evaluate the feasibility of using ultrasonography to preoperatively predict the autologous hamstring graft diameter for anterior cruciate ligament (ACL) reconstruction in the Zhuang population and determine a reliable measurement level using ultrasound.

**Methods:** Twenty-four Zhuang patients who were scheduled for ACL reconstruction using four-strand semitendinosus tendon (ST) and gracilis tendon (G) (4S-STG) autografts were included in this study. Ultrasonographic examinations of the ST and the G on the damaged side were conducted before the operation. We recorded the transverse diameter (TD), anterior–posterior diameter (APD), cross-sectional area (CSA), and perimeter (P) of the tendons. The measurements were obtained from two levels of the tendons: the widest point of the medial femoral epicondyle (level 1) and the myotendinous junction of the sartorius (level 2). We also calculated the combined (ST + G) TD, APD, CSA, and *p* values. Then, we obtained the intraoperative measurements. The correlation between the ultrasonic and intraoperative measurements was analyzed, and the advantages of the ultrasonic measurements at the two different levels were compared.

**Results:** When we measured at level 1, we found that part of the ultrasonic measurements were correlated with intraoperative measurements. The preoperative CSA of the G (P-GCSA) can be used to distinguish a 4S-STG autograft diameter of ≥8 mm (*p* < 0.01, mean difference = 3.7). The area under the P-GCSA curve was 0.801 (*p* < 0.05). A P-GCSA of 8.5 mm^2^ could be used to predict a 4S-STG autograft diameter of ≥8 mm with a sensitivity of 61.1% and specificity of 83.3%. However, there was no correlation between the ultrasonic and intraoperative measurements at level 2.

**Conclusion:** Preoperative ultrasound can be used to predict the sufficient diameter of 4S-STG autografts when considering patients from Zhuang who are undergoing ACL reconstruction. The ultrasonic measurement should be obtained at the widest point of the medial femoral epicondyle.

## Introduction

An anterior cruciate ligament (ACL) rupture is a common sports injury of the knee joint. According to statistics, approximately 350,000 people in the United States and more than one million people worldwide undergo ACL reconstruction every year (Tiamklang et al., 2012; Sugimoto et al., 2016; Davies et al., 2017). There are many graft options for ACL reconstruction, including autologous grafts of bone–patellar tendon–bone (BPTB), the hamstring tendon, the quadriceps femoris tendon, and the peroneal long tendon. Research (Li et al., 2011) shows that the maximum load of the four-strand semitendinosus and gracilis tendon (4S-STG) autograft is greater than that of the ACL, and its biomechanical performance exceeds even that of BPTB grafts. There are fewer complications in the donor area, such as anterior knee pain, knee extension disorder, and other serious complications (Wipfler et al., 2011). At present, arthroscopic hamstring tendon transplantation for ACL reconstruction is the main surgical method (Chen et al., 2017), and most scholars advocate the use of an autologous tendon graft for a patient’s first ACL reconstruction (Wang et al., 2018).

Although the graft size parameters for successful ACL reconstruction have not been clearly defined, the problem of insufficient graft size is occasionally found. Generally, it is believed that the strength of grafts with diameters of <7 mm is insufficient to reconstruct the ACL, and the larger the diameter, the greater the strength (Asif et al., 2016; Ho et al., 2016). However, investigations have shown that grafts of <8 mm in diameter have a higher failure rate in terms of ACL reconstruction (Magnussen et al., 2012). Because the semitendinosus tendon (ST) and gracilis tendon (G) vary in length and thickness due to individual differences, shorter and thinner tendons are not conducive to multiple ligament folding and weaving and multi-bundle reconstruction. Therefore, it is necessary to establish a simple and standardized preoperative measurement method to predict the size of grafts by preoperatively screening patients at risk of insufficient graft diameter from autologous hamstring tendon transplantation.

Most researchers (Wernecke et al., 2011; Beyzadeoglu et al., 2012; Erquicia et al., 2013; Galanis et al., 2016; Corey et al., 2018; Hollnagel et al., 2019; Vardiabasis et al., 2019; OlivaMoya et al., 2020; Heijboer et al., 2021; Partan et al., 2021) have studied the feasibility of using preoperative magnetic resonance imaging (MRI) to predict intraoperative graft diameters. Although OlivaMoya et al. (2020) consider this is unfeasible, others believe that MRI can be used to predict the final intraoperative graft diameter. However, due to the high cost and time-consuming nature of MRI, and because the technique is unsuitable for examining some patients, there is an urgent need to find a simpler and more effective method for preoperative evaluation.

Ultrasound examination has the advantages of low cost, high safety, no radioactivity, easy data access, and repeatability. In recent years, researchers (Erquicia et al., 2013; Galanis et al., 2016; Rodriguez-Mendez et al., 2017; Astur et al., 2018; MohdAsihin et al., 2018; Momaya et al., 2018; Sumanont et al., 2019; Takenaga et al., 2019) have studied the feasibility of preoperative evaluation by ultrasound. All the research subjects were patients with a primary ACL injury who needed autologous 4S-STG autograft transplantation. The research population included patients from Europe, America, South America, and Asia (Malaysia and Thailand). However, there have been no reports for the Chinese population. In addition, there are differences in the selection of patient position and tendon measurement level, e.g., Takenaga et al. (2019) and Sumanont et al. (2019) patients assumed a supine position, while other studies mostly selected the prone position. Takenaga et al. (2019) believed that the ST, G, and the myotendinous junction of the sartorius could be displayed on the same ultrasonic plane. Therefore, they measured the ST and G at the level of the myotendinous junction of the sartorius using ultrasound. Most of the other studies followed the same plan as the MRI studies (Erquicia et al., 2013; Galanis et al., 2016), with the measurement level of the tendon between the joint line and the medial femoral epicondyle (Erquicia et al., 2013; Galanis et al., 2016; Rodriguez-Mendez et al., 2017; Astur et al., 2018; MohdAsihin et al., 2018; Momaya et al., 2018).

Studies have emphasized the feasibility of using ultrasound for predicting the size of the final 4S-STG autograft for ACL reconstruction (Erquicia et al., 2013; Galanis et al., 2016; Rodriguez-Mendez et al., 2017; MohdAsihin et al., 2018; Sumanont et al., 2019; Takenaga et al., 2019). However, some researchers (Astur et al., 2018; Momaya et al., 2018) hold the opposite view that preoperative ultrasound is unreliable for predicting the diameter of intraoperative grafts. When studying the feasibility of preoperative evaluation with ultrasound, standardizing the examination method and selecting a reliable ultrasonic measurement level may make the results more realistic.

Therefore, since they had not been studied previously, we selected the Zhuang nationality (the largest minority population in China) as research objects. We compared the advantages of the two commonly used ultrasonic measurement levels reported in the literature to explore the value of preoperative ultrasound for predicting 4S-STG autograft diameters in the Zhuang population using a more reliable measurement method.

## Methods and measurements

This study was performed in the People’s Hospital of Guangxi Zhuang Autonomous Region between May 2020 and May 2021. Our study included 24 patients, including 13 males and 11 females, with an average age of 33.7 ± 8.4 years. All patients were from the Zhuang population, they were 18 years or older, and they underwent primary ACL reconstruction using a 4S-STG autograft of the ipsilateral leg as donors in the Department of Orthopedics. Patients with a history of ACL injury, knee injury, previous knee surgery (except for diagnostic arthroscopy), or 5S-STG and 6S-STG grafts were excluded. Patients who had disabling complications, radiographically altered osteoarthritis, morbid obesity (i.e., a body mass index [BMI] of >40 kg/m^2^), or who were severely underweight (i.e., a BMI of <18 kg/m^2^) were also excluded from the study.

### Ultrasonic measurement

**FIGURE 1:**
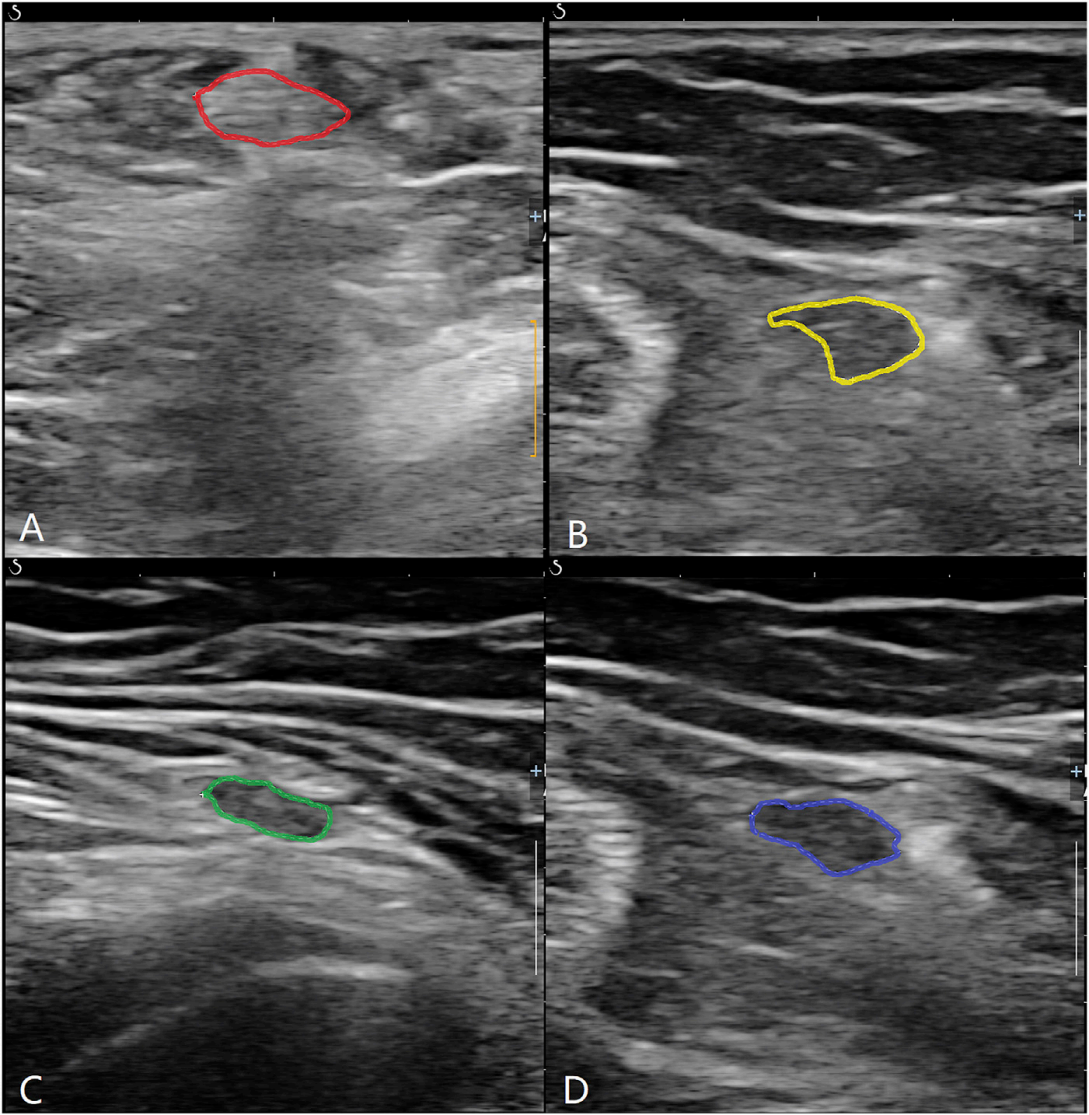
ST and G were identified at two levels. **(A)** ST at lever 1; **(B)** ST at level 2; **(C)** G at level 1; **(D)** G at level 2.

The study used an Aixplorer®acoustic blue Doppler ultrasound system with a high-frequency probe of 15–4 MHz. Musculoskeletal scanning conditions were selected. Patients assumed a prone position with straight legs. The ST and G on the damaged side were identified at two levels: the widest point of the medial femoral epicondyle (level 1) and the myotendinous junction of the sartorius (level 2) (Figure 1). At each level, the transverse diameter (TD), anterior–posterior diameter (APD), cross-sectional area (CSA), and perimeter (P) of each tendon were measured on a short-axis plane of double-magnification images. The combined TD, APD, CSA, and P of the two tendons were calculated. Each tendon’s CSA was measured by manual tracing. All data were measured three times, and the mean value of the three measurements was taken as the final value. All ultrasound measurements were performed by the same senior attending physician with more than 5 years of musculoskeletal ultrasonic experience.

### Intraoperative measurement

**FIGURE 2:**
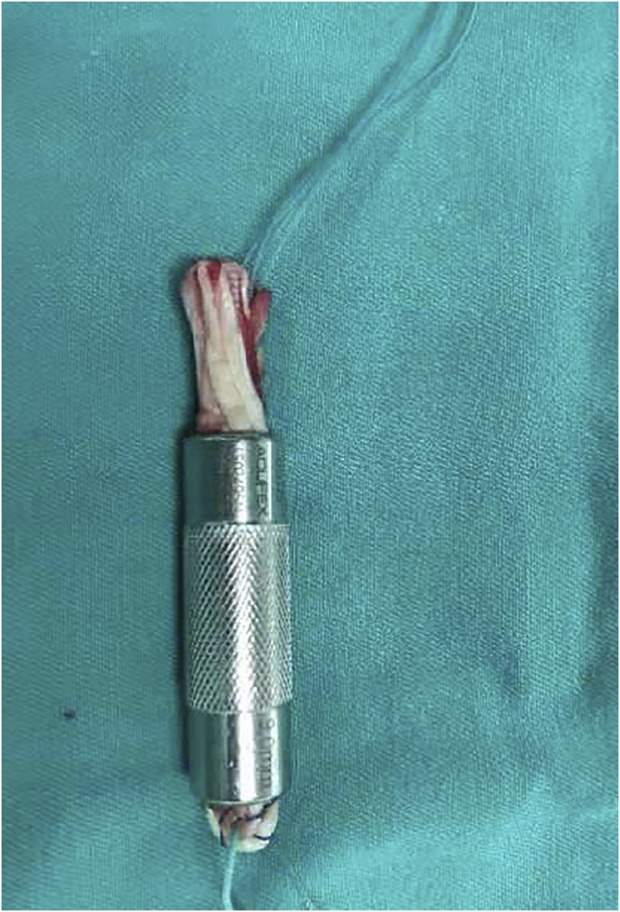
Intraoperative measurement of the final 4S-STG diameter.

A 3-cm oblique skin incision was made at the medial side of the tibial tubercle, and the pes anserinus was exposed through blunt dissection. The ST and G were identified and harvested individually using a tendon stripper after the sartorius tendon membrane was split by an L-shaped incision. The harvested tendons were cleaned of any extra tissue on a graft preparation table. The length (L) and diameter (D) of each tendon were measured. The two tendons were duplicated to form a bundle of four layers as the final 4S-STG autograft. Then, the graft was passed through a slotted measurement module (the measuring device of Smith and nephew company, its diameter interval was 1 mm) for sizing, and its diameter was recorded (Figure 2). The intraoperative combined diameter (ICD) was the addition of the intraoperative diameter of the ST and G.

### Statistical analysis

The Paired Samples *t* Test was used to compare the ultrasonic measured differences of levels 1 and 2. The Pearson correlation coefficient was used to analyze the relationship between the ultrasonic and intraoperative measurements. Independent Samples *t* Test was used to investigate whether there is a statistical difference between the two groups in the ultrasonic measurements corresponding to the graft diameter (<8 mm and ≥ 8 mm).

The ability of the ultrasonic measurements to predict a final 4S-STG autograft diameter of not less than 8 mm was analyzed using a receiver operating curve, and the cutoff point was obtained according to the Youden index. A *p* value below 0.05 was considered significant. All statistical analyses were performed using SPSS version 22 software.

Approval was obtained from the ethics committee of the People’s Hospital of Guangxi Zhuang Autonomous Region, and written informed consent was obtained from patients prior to the study.

## Results

The ultrasonic measured values from level 1 and level 2 are shown in Table 1. There were no significant differences between level 1 and level 2 in terms of the preoperative TD of the ST (P-STTD) (t = 0.738,*p* = 0.468), the preoperative APD of the ST (P-STAPD) (t = 1.607,*p* = 0.122), the preoperative CSA of the ST (P-STCSA) (t = 1.835,*p* = 0.079), the preoperative P of the ST (P-STP) (t = 1.146,*p* = 0.264). The values for the two levels in terms of the preoperative APD of the G (P-GAPD) (t = 3.823,*p* = 0.001), the preoperative CSA of the G (P-GCSA) (t = 4.895,*p* = 0.000), and the preoperative P of the G (P-GP) (t = 3.542,*p* = 0.002) had statistical differences, while there was no significant differences between the two levels in terms of the preoperative TD of the G (P-GTD) (t = 2.007,*p* = 0.057).

**TABLE 1:** Ultrasonic measurements at level 1 and level 2 (mm).

	STTD	STAPD	STCSA	STP	GTD	GAPD	GCSA	GP
Level 1	6.10 ± 2.05	3.07 ± 0.51	15.75 ± 7.27	16.25 ± 4.46	5.05 ± 1.52	2.34 ± 0.98^a^	9.96 ± 4.18^a^	13.54 ± 3.36^a^
Level 2	5.72 ± 1.96	2.86 ± 0.67	12.67 ± 5.55	15.09 ± 3.89	4.53 ± 1.19	1.75 ± 0.48	7.25 ± 2.67	11.95 ± 3.09

Abbreviation: ST, semitendinosus tendon; G, gracilis tendon; TD, transversediameter; APD:anterior posterior diameter; CSA, cross-sectional area; P, perimeter.

a *p*< 0.05.

The correlation between the ultrasonic measurements at level 1 and the intraoperative measurements is shown in Table 2. The TD of the tendons was not related to the intraoperative measurements. There was no relationship between the ultrasonic measurements at level 2 and the intraoperative measurements.

**TABLE 2:** Correlation between Ultrsound and Intraoperative measurements^a^.

	ISTL	ISTD	IGL	IGD	ICD
P-STTD	—	—	—	—	—
P-STAPD	0.640^b^	0.530^b^	—	—	0.576^b^
P-STCSA	—	—	—	—	—
P-STP^*^	—	0.422^c^	—	—	0.418c
P-GTD	—	—	—	—	—
P-GAPD	—	—	—	—	—
P-GCSA	—	—	—	—	0.440^c^
P-GP^*^	—	—	—	—	—
P-CTD	—	—	—	—	—
P-CAPD	—	—	—	—	—
P-CCSA	—	—	—	—	0.414^c^
P-CP^*^	—	—	—	—	0.445^c^

Abbreviation: ST, semitendinosus tendon; G, gracilis tendon; P, preoperative; TD, transverse diameter; APD:anterior posterior diameter; CSA, cross-sectional area; P*, perimeter; I, intraoperative; L, lengths; C, combined; 4S-STG, 4-strand semitendinosus and gracilis tendon autograft.

a Ultrsound measurements at level 1.

b *p* < 0.01.

c *p* < 0.05.

When the 4S-STG autograft diameter was ≥ 8 mm, the average P-GCSA at level 1 was 10.9 mm^2^. When the 4S-STG autograft diameter was <8 mm, the average P-GCSA at level 1 was 7.2 mm^2^ (t = 3.110, *p* = 0.005, Mean Difference = 3.7). There was no significant difference for the rest of ultrasonic measurements at level 1 and level 2 to distinguish a 4S-STG autograft diameter of ≥8 mm.

**FIGURE 3:**
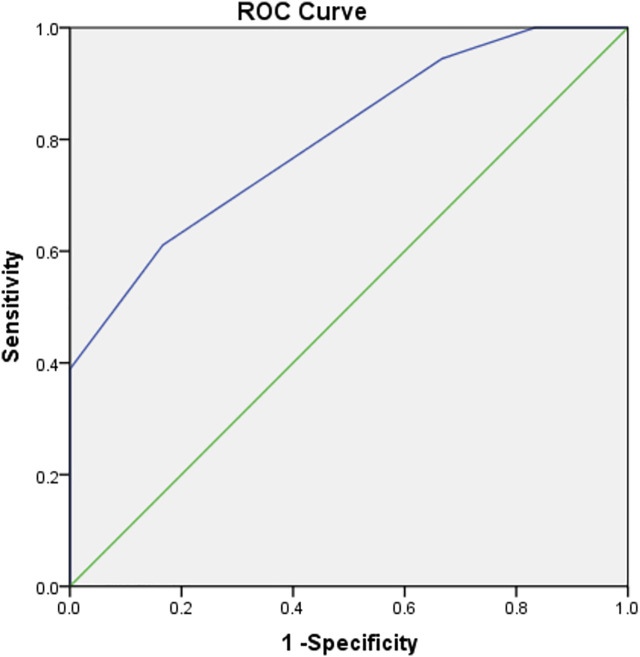
The area under the curve of P-GCSA.

The area under the P-GCSA curve was 0.801 (*p* < 0.05). A P-GCSA of 8.5 mm^2^ could be used to predict a 4S-STG autograft diameter of ≥8 mm with a sensitivity of 61.1% and a specificity of 83.3% (Figure 3; Supplementary Appendix S1).

## Discussion

By studying the patients from Zhuang who were scheduled to undergo ACL reconstruction using 4S-STG autografts, we determined the value of using preoperative ultrasound to predict the final graft diameter. The important findings of this study are as follows: 1) In this group of patients from Zhuang, the P-GCSA at level 1 can distinguish the final graft diameter with 8 mm as the dividing point. The P-GCSA could be used to predict a sufficient final 4S-STG autograft diameter. 2) In this group of cases, the ultrasonic measurements at level 1 were correlated with the intraoperative measurements, and P-GCSA at level 1 can be used to distinguish and predict a 4S-STG autograft diameter of ≥8 mm, while those at level 2 were not. Therefore, we considered it is more reliable to select the widest point of the medial femoral epicondyle as the tendon measurement level for preoperative evaluation.

To our knowledge, this study was the first to use people from Zhuang as a research population. Our results will provide a method to predict the final 4S-STG diameter for patients from Zhuang who are about to undergo an initial autologous hamstring tendon transplantation for ACL reconstruction and preoperatively screen patients at risk of an insufficient graft diameter. This will help formulate a reasonable preoperative plan and arrange appropriate alternative transplantation sources and patient consultations related to graft selection. In addition, our study compared the ultrasonic measurement of different planes for the first time, which is helpful for selecting a more reliable ultrasonic measurement level and generating more realistic results.

According to previous studies (Erquicia et al., 2013; Galanis et al., 2016; Rodriguez-Mendez et al., 2017; Astur et al., 2018; MohdAsihin et al., 2018; Momaya et al., 2018; Takenaga et al., 2019), we identified two ultrasonic measurement levels commonly used by scholars: the widest point of the medial femoral epicondyle (level 1) and the myotendinous junction of the sartorius (level 2). Then, we compared the significant differences between the two levels of ultrasonic measured values. The research revealed significant differences between the two levels in terms of P-GCSA, P-GAPD and P-GP, i.e., in this group of patients, there was little change in the shape and size of the ST at the two levels, while the shape and size of the G at level 2 was flatter and smaller than at level 1.

The Pearson correlation coefficient showed no correlation between the ultrasonic measurements at level 2 and the intraoperative measurements. However, some of the ultrasonic measurements at level 1 were correlated with the intraoperative measurements. There is no significant statistical difference between some measured values of ultrasonic measurement at level 1and level 2. The author believes that the possible reasons are that the variables investigated in the two results are not completely consistent, and the sample size of this study is small, which needs to be further discussed by increasing the sample size. In addition, the results showed that P-GCSA at level 1 can be used to distinguish and predict a 4S-STG autograft diameter of ≥8 mm, while those at level 2 were not. Therefore, we believe that level 1 is more reliable for preoperative evaluation than level 2. This may be attributed to the following reasons: 1. Level 1 is closer to the proximal end of the body, and the tendon is relatively thick; this makes it easier to identify and reduces the measurement error caused by the unclear identification of the tendon boundary 2. The myotendinous junction of the sartorius was used at level 2, which was more subjective than the observation level of bone markers at level 1.

Takenaga et al. (2019) reported that the CSA of the G, ST, and (ST + G) correlated with the diameters of 2G (*r* = 0.464, *p* = 0.039), 2ST (*r* = 0.712, *p* < 0.001), and 4STG (*r* = 0.792, *p* < 0.001), respectively. Other researchers considered that the measured combined CSA (ST + G) and 4S-STG autograft diameter (*p* = 0.023) were statistically significantly correlated (MohdAsihin et al., 2018). Another study (Rodriguez-Mendez et al., 2017) reported that the diameter of the gracilis tendon (GRd) had a positive correlation with the diameter of the semitendinosus tendon (SMTd), the SMT length, the GRd, the SMTd + GRd, and the final tendon length. The SMTd positively correlated with both the SMT length and the final tendon length, while the SMTd + GRd correlated with multiple transoperative tendon lengths and diameters.

In the present research, we measured the TD, APD, CSA, and P of the tendons using ultrasound. The APD, CSA, and P were related to all the intraoperative measurements except TD. We concluded that the P-STAPD and P-STP had a positive correlation with the intraoperative diameter of the ST and the ICD. In addition, the P-STAPD was positively correlated with the intraoperative length of the ST. However, for the G, only the P-GCSA was positively correlated with the ICD. The preoperative combined CSA (P-CCSA) and the P (P-CP) were positively correlated with the ICD. The results are both similar to and different from those reported by previous scholars (Galanis et al., 2016; Rodriguez-Mendez et al., 2017; Astur et al., 2018; MohdAsihin et al., 2018; Momaya et al., 2018; Sumanont et al., 2019; Takenaga et al., 2019). Possible reasons include different study populations and inconsistent ultrasonic and intraoperative measurement indices and methods.

In previous studies, some researchers (Erquicia et al., 2013; MohdAsihin et al., 2018; Takenaga et al., 2019) have reported that combined CSAs were used to predict the diameter of the 4S-STG autograft. For example, Rodriguez-Mendez et al. (2017) believed that the diameter of the G (measured by ultrasound at 4.5 mm) could predict high-risk patients with a final 4S-STG autograft diameter of <8 mm. Another study showed that an ST CSA of 16 mm^2^ could be used as a cutoff point to predict a diameter for a 4S-STG of ≥8 mm (Sumanont et al., 2019). In the present study, we found that the area under the P-GCSA curve at level 1 reached 0.801; i.e., the larger the P-GCSA at level 1, the larger the actual graft diameter. P-GCSA at level 1 was of specific value in predicting a sufficient final 4S-STG autograft diameter. When the lower limit was 8.5 mm^2^, a final 4S-STG autograft diameter of no less than 8 mm could be predicted. The sensitivity and specificity were 61.1% and 83.3%, respectively.

The limitation of this study is that the number of cases was small, and it will be necessary to increase the sample size in further studies. In addition, the slotting module we used to measure the final graft diameter had diameter intervals of 1 mm, which were slightly less accurate than intervals of 0.5 mm.

## Conclusion

For the patients from Zhuang who were undergoing ACL reconstruction with autologous hamstring tendon transplantation, the P-STAPD, P-STP, P-GCSA, P-CCSA, P-CP, and P-CAPD obtained at level 1 (the widest point of the medial femoral epicondyle) were correlated with the intraoperative measurements. However, only theP-GCSA can be used to distinguish a 4S-STG autograft diameter of ≥8 mm. Furthermore, when 8.5 mm^2^ was used as the cutoff point for the P-GCSA, it could be used to predict a final 4S-STG autograft diameter of ≥8 mm.

## Data Availability

The original contributions presented in the study are included in the article/Supplementary Material, further inquiries can be directed to the corresponding author.

## Abbreviations

4S-STG autograft, 4-strand semitendinosus and gracilis tendon autograft; ACL, anterior cruciate ligament; APD, anterior posterior diameter; CSA, cross-sectional area; D, diameters; G, gracilis tendon; ICD, intraoperative combined diameter; IGD, intraoperative diameter of G; IGL, intraoperative length of G; ISTD, intraoperative diameter of ST; ISTL, intraoperative length of ST; L, lengths; MRI, magnetic resonance imaging; P, perimeter; P-CAPD, preoperative combined APD; P-CCSA, preoperative combined CSA; P-CP, preoperative combined P; P-CTD, preoperative combined TD; P-GAPD, preoperative APD of G; P-GCSA, preoperative CSA of G; P-GP, preoperative P of G; P-GTD, preoperative TD of G; P-STAPD, preoperative APD of ST; P-STCSA, preoperative CSA of ST; P-STP, preoperative P of ST; P-STTD, preoperative TD of ST; ROC, receiver operating curve; ST, semitendinosus tendon; TD, transverse diameter
