# Supplementary material for: Predicting autologous hamstring graft diameter and finding reliable measurement levels in the Zhuang population using preoperative ultrasonography
Source: Front Physiol. 2022 Aug 24;13:916438. doi: 10.3389/fphys.2022.916438 (PMC9448865; doi:10.3389/fphys.2022.916438)
Supplement: Supplementary file 1 [file Table1.DOCX]

Coordinates of the curve

Test result variable(s): P-GCSA at level (mm2)

| Positive if Greater Than or Equal To^a^ | Sensitivity | 1-Specifity |
| --- | --- | --- |
| 4.0000 | 1.000 | 1.000 |
| 5.5000 | 1.000 | 0.833 |
| 6.5000 | 0.944 | 0.667 |
| 7.5000 | 0.833 | 0.500 |
| 8.5000 | 0.611 | 0.167 |
| 9.5000 | 0.389 | 0.000 |
| 11.0000 | 0.333 | 0.000 |
| 13.5000 | 0.278 | 0.000 |
| 16.0000 | 0.167 | 0.000 |
| 17.5000 | 0.111 | 0.000 |
| 19.5000 | 0.056 | 0.000 |
| 22.0000 | 0.000 | 0.000 |

The test results variable (s):P-GCSA at level 1 (mm2) has at least one tie between the positive actual state group and the negative actual state group.

1. The smallest cutoff value is the minimum observed test value minus 1, and the largest cutoff value is the maximum observed test value plus 1; All the other cutoff values are the averages of two consecutive ordered observed test values.
